# A high-fat diet promotes depression-like behavior in mice by suppressing hypothalamic PKA signaling

**DOI:** 10.1038/s41398-019-0470-1

**Published:** 2019-05-10

**Authors:** Eirini Vagena, Jae Kyu Ryu, Bernat Baeza-Raja, Nicola M. Walsh, Catriona Syme, Jonathan P. Day, Miles D. Houslay, George S. Baillie

**Affiliations:** 1 Gladstone Institute of Neurological Disease, University of California, San Francisco, CA 94158 USA; 2 College of Veterinary, Medical and Life Sciences, Institute of Cardiovascular and Medical Sciences, University of Glasgow, Glasgow, Scotland G12 8QQ UK; 3 Institute of Pharmaceutical Science, King’s College London, London, England SE1 9NH UK

**Keywords:** Molecular neuroscience, Physiology, Clinical genetics

## Abstract

Obesity is associated with an increased risk of depression. The aim of the present study was to investigate whether obesity is a causative factor for the development of depression and what is the molecular pathway(s) that link these two disorders. Using lipidomic and transcriptomic methods, we identified a mechanism that links exposure to a high-fat diet (HFD) in mice with alterations in hypothalamic function that lead to depression. Consumption of an HFD selectively induced accumulation of palmitic acid in the hypothalamus, suppressed the 3′, 5′-cyclic AMP (cAMP)/protein kinase A (PKA) signaling pathway, and increased the concentration of free fatty acid receptor 1 (FFAR1). Deficiency of phosphodiesterase 4A (PDE4A), an enzyme that degrades cAMP and modulates stimulatory regulative G protein (Gs)-coupled G protein-coupled receptor signaling, protected animals either from genetic- or dietary-induced depression phenotype. These findings suggest that dietary intake of saturated fats disrupts hypothalamic functions by suppressing cAMP/PKA signaling through activation of PDE4A. FFAR1 inhibition and/or an increase of cAMP signaling in the hypothalamus could offer potential therapeutic targets to counteract the effects of dietary or genetically induced obesity on depression.

## Introduction

Obesity predominantly develops in response to increased consumption of energy-dense diets and a sedentary lifestyle^1^. Rare genetic mutations in the central melanocortin pathway are responsible for the development of monogenic obesity in humans^2^. The main clinical consequences of obesity are abnormalities characteristic of the metabolic syndrome (e.g., hypertension, insulin resistance, or dyslipidemia) and an increased risk of diseases such as cancer^3,4^. Furthermore, obesity has been linked to depression^5,6^, with both epidemiological and clinical studies demonstrating a positive association between these two disorders^7^. Nonetheless, the precise mechanism underlying the interaction between obesity and depression has yet to be elucidated.

Although the neuropathophysiology of depression remains unclear, abnormalities in monoamine signaling components, such as serotonin and dopamine, have been implicated in the development of this condition^8^. Clinical observations around mid-90s suggested that depression results from decreased monoamine function in the brain^8^. Some of the key drugs currently used to treat depression target monoamine signaling^8^; however, not all patients benefit from such intervention^9^. The presence of obesity, or overweight, places patients with major depression at risk of resistance to the antidepressant fluoxetine, regardless of the severity of depression at baseline^10^. When compared with patients of normal body weight, overweight and obese patients showed a substantially slower response to antidepressant treatment, less improvement in neuroendocrinology and cognitive processing, and less antidepressant-induced weight gain^11^. This observation suggests the involvement of unique pathways for depression in the overweight and obese population.

The neurocircuitry of depression is complex and involves portions of the limbic system, such as hippocampus, amygdala, thalamus, cortex, and hypothalamus^12^. From all these brain regions that play a crucial role in depression, hypothalamus is the main regulator of energy homeostasis, located in a region highly vascularized with ample communication with the periphery, and has been implicated in both obesity and depression^13^. Signaling via 3′, 5′-cyclic AMP (cAMP) appears to have a key role in the pathophysiology and pharmacology of depression^14^. Even though the mechanism of action of antidepressants is very complex and not well understood, it is believed that antidepressant treatments involve adaptations of the cAMP signaling cascade^15^. Generation of cAMP by adenylyl cyclase activity occurs after stimulation of the G protein-coupled receptors (GPCRs). Antidepressants often increase coupling of stimulatory G proteins with adenylyl cyclases^16^, thereby increasing both the activity of cAMP-dependent protein kinase A (PKA)^17^ and the expression and function of cAMP response element-binding protein (CREB)^18^. Protein phosphorylation by PKA regulates a vast variety of neuronal functions^19^.

In depression signaling via cAMP may be impaired by cyclic nucleotide phosphodiesterases (PDEs), which provide the sole route for cAMP degradation in cells^20^. Of all the different PDEs, members of the PDE4 gene family play a major role in regulating cognition and depressive disorders^21^. The PDE4 gene family (*PDE4A, PDE4B, PDE4C*, and *PDE4D*) gives rise to >20 different isoforms^22^. PDE4C is the only one not expressed in brain according to previous studies^23^. Although much of the PDE4 sequence is conserved between isoforms, the unique N-terminal region confers direct isoform-specific targeting to intracellular signaling complexes^24^ and interaction with anchor/scaffold proteins^25^, allowing the fine tuning of cAMP signaling to discrete subcellular locations and specific pathways^26^.

The most important neuronal pathway for human obesity is the central melanocortin signaling pathway, as the majority of genes responsible for human monogenic obesity are components of this pathway^2^. The central melanocortin pathway is regulated by dietary fatty acids^27,28^, which bind to different fatty acid receptors, a subfamily of the GPCRs superfamily, to convey intracellular signaling pathways^29^. There are four main free fatty acid (FFA) receptor divisions according to the length and saturation of fatty acids they bind to: FFA receptor 1 (FFAR1 also known as GPR40) that binds medium and long chain saturated fatty acids such as palmitic acid^30^, FFA receptor 3 (FFAR3 also known as GPR41) and FFA receptor 2 (FFR2 also known as GPR42) that both bind short chain fatty acids^31^, and finally the FFA receptor 4 (FFAR4 also known as GPR120) that binds ω-fatty acids^32^. Just as PDEs may have a mechanistic role in the development of depression, they may also influence the development of obesity. Members of the PDE4 family can interact with GPCRs^33^ via β-arrestin proteins, which act as scaffolds to localize PDE4s to ligand-activated GPCRs^34,35^.

Even though a positive association between obesity and depression has been established, which of the two plays a causative role for the development of the other one and what is the molecular mechanism(s) of this phenomenon remains unknown. In the present study, we found that either dietary or genetically induced obesity (GIO) in mice lead to depression phenotype and this phenomenon occurs via the disruption of the cAMP/PKA signaling pathway. Furthermore, we identified that loss of *PDE4A* can prevent both dietary and genetically induced depression-like behavior phenotype in mice. In addition, we found that the consumption of a fat-dense diet leads to an influx of dietary fatty acids specifically in the hypothalamus. These fatty acids can directly modulate the PKA signaling pathway that is responsible for the development of depression. These findings suggest that the influx of saturated fatty acids due to the consumption of an high-fat diet (HFD) can alter the cAMP/PKA signaling cascade and that result in the development of depression phenotype.

## Results

### Dietary-induced obesity (DIO) is accompanied by a depression-like phenotype in mice

**Fig. 1:**
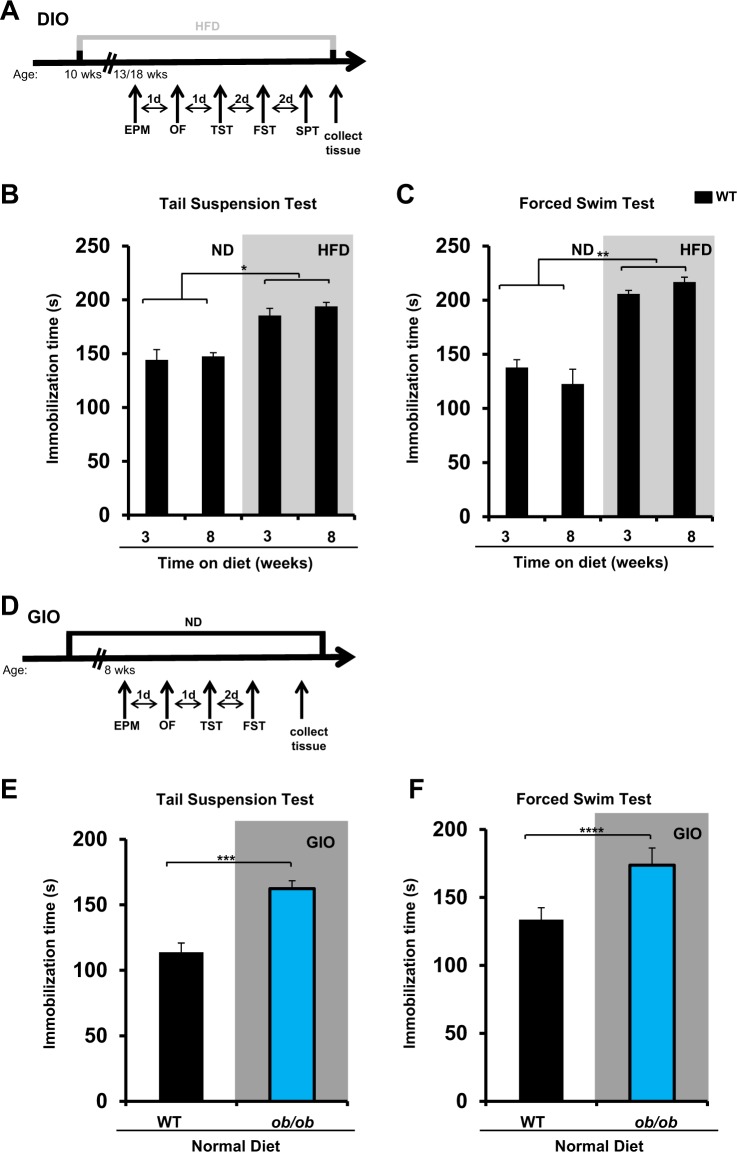
Dietary or genetically induced obesity is accompanied by a depression-like phenotype in mice. **a** Schematic of the experimental plan for dietary-induced obesity (DIO) and a series of behavioral tests (EPM elevated plus maze, FST forced swim test, HFD high-fat diet, ND normal diet, OF open field, SPT sucrose preference test, TST tail suspension test). **b** TST and **c** FST for aged-matched wild-type (WT) C57BL/6J mice maintained for a period of 3 weeks or 8 weeks on either ND or HFD (*n* = 10 per group, experiment repeated twice; *P < 0.05, **P < 0.01 by linear mixed model fit by restricted maximum likelihood (REML). **d** Schematic of the experimental plan for genetically induced obesity (GIO) and a series of behavioral tests. **e** TST and **f** FST for wild-type (WT) C57BL/6J and *ob/ob* mice maintained on a ND for a period of 12–16 weeks (*n* = 8–10 per group, experiment repeated twice; **P < 0.01, ****P < 0.0005 by unpaired Student's *t*-test)

To determine whether the consumption of a fat-dense diet plays a causative role in the development of depression, we first examined depression-related behaviors among mice fed a HFD for 3 or 8 weeks (Fig. 1a), where 60% of caloric intake is derived from fat. Induction of depression-like behavior, as assessed by increased immobilization time during the tail suspension and forced swim tests, was observed after just 3 weeks and persisted at 8 weeks (Fig. 1b, c). Consumption of an HFD was also accompanied by the consumption of less sucrose solution than was observed for wild-type (WT) aged-matched control mice maintained on a normal diet (ND), a test related to anhedonia (Supplementary Fig. S1A), a characteristic feeling of depressed patients that describes their inability to experience pleasure by enjoyable activities.

As expected, mice fed an HFD gained substantially more weight than the control mice fed ND, even from the first week of the intervention (Supplementary Fig. S1B). Increased body weight did not correlate with increased immobilization during the tail suspension and forced swim tests after 3 weeks (Supplementary Fig. S1C), suggesting that the performance of the mice in these tests was not affected by their increased body weight. In agreement with that, the depression-like phenotype developed on mice fed an HFD was not accompanied by less locomotor or rearing activity during the open field test compared with mice on ND (Supplementary Fig. S2A).

These results suggest that consumption of an HFD can contribute to the development of depression-like behavior.

### GIO is accompanied by a depression-like phenotype in mice

To determine whether GIO also results in the depression-like phenotype, we conducted the behavioral tests with the leptin-deficient mice (*ob/ob*), which develop obesity from the third week of age even when maintained on an ND (Fig. 1d). During both the tail suspension and forced swim tests, the immobilization time was greater in 8-week-old *ob/ob* mice than in WT aged-matched mice (Fig. 1e, f). As expected, even from the third week of life, *ob/ob* mice on an ND gained significantly more weight than WT mice on an ND (Supplementary Fig. S2B). Even though the DIO did not affect the locomotor activity of mice measured by the open field test, the *ob/ob* mice had less locomotor and rearing activity compared with their WT aged-matched control mice (Supplementary Fig. S2A).

These results suggest that like DIO, GIO promotes the development of a depressive-like phenotype in mice.

### DIO alters gene expression profiles in the hypothalamus

Given the early onset of the depression-like phenotype in the group of mice fed an HFD, which did not correlate with body weight, we hypothesized that consumption of an HFD alters the molecular signaling pathways in the hypothalamus, which is a brain region with major role in the control of both obesity and depression^36^. We used genome-wide microarray analysis to determine the hypothalamic gene expression profile of WT mice fed an ND versus WT mice fed an HFD for a period of 4 or 8 weeks.

**Fig. 2:**
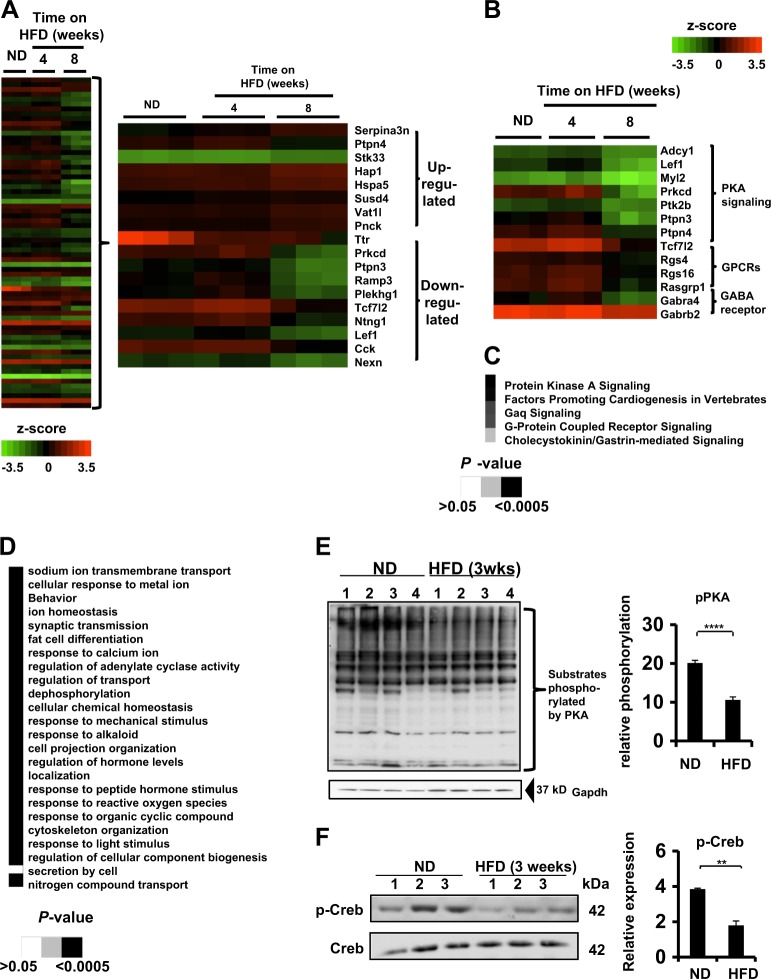
Dietary-induced obesity alters gene expression profiles in the hypothalamus. **a** (Left) Global gene expression analysis of hypothalamic samples taken from wild-type (WT) C57BL/6J mice maintained on either a normal diet (ND) or a high-fat diet (HFD) for a period of 4 or 8 weeks. Colors represent values as log_10_ after normalization by *z*-score. Low expression level is shown in green and high expression level in red. Each column represents a different mouse and in each condition there are three different mice. (Right) The most upregulated and downregulated genes affected by HFD compared with ND. **b** Heat map of the key genes altered by HFD and are involved in the three main signaling pathways affected by the consumption of an HFD. Colors represent values as log_10_ after normalization by *z*-score. **c** Microarray analysis by IPA Ingenuity Pathway Analysis software of the canonical pathways affected by HFD compared with ND. Grayscale represents *P*-values of the statistics by the IPA Ingenuity pathway analysis. **d** Gene ontology (GO) enrichment pathways that were affected by the consumption of an HFD compared with ND. Grayscale represents the *P*-values statistics of each pathway by GO. **e** (Left) Western blot analysis detecting the phosphorylated status of protein kinase A (PKA) substrates of whole-hypothalamic homogenates from WT C57BL/6J mice fed either ND or HFD for 3 weeks. pPKA refers to the sum of all phosphorylated substrates of PKA. GAPDH was used as a loading control. (Right) Quantification of the total phosphorylated status of PKA substrates between whole-hypothalamic homogenates from mice fed either ND or HFD. Each line represents a different mouse. (*n* = 4 mice per group, ****P < 0.0001 by two-tail unpaired Student's *t*-test). **f** (left) Western blot analysis of total phosphorylated cAMP response element-binding protein (p-Creb) and cAMP response element-binding protein (CREB) protein levels in whole-hypothalamic homogenates of mice fed either ND or HFD for 3 weeks. (Right) Quantification of the total phosphorylated levels of Creb between whole-hypothalamic homogenates from mice fed either ND or HFD for 3 weeks. Each line represents a different individual mouse. (*n* = 3 mice per group, **P < 0.01 by two-tail unpaired Student's *t*-test). All data in the figure are represented as mean ± SEM

A total of 68 genes exhibited altered expression patterns in the hypothalamus of mice fed an HFD for 8 weeks compared with mice fed an ND, with false discovery rate (FDR) < 0.05 (Fig. 2a). Moreover, the most highly significant upregulated and downregulated genes affected by the consumption of a HFD are shown (Fig. 2a). The PKA signaling was the most affected pathway upon the consumption of HFD for 8 weeks (*P* = 0.0000398) (Fig. 2b, c). Genes regulating the PKA signaling pathway were significantly decreased after 8 weeks on an HFD (Table 1). Other pathways were also suppressed, including the GPCR signaling nodes and GABA receptor signaling pathways, which are involved in neuronal functions (Fig. 2b, c). Gene ontology (GO) enrichment analysis revealed that the hypothalamic adenylate cyclase pathway, a major contributor in the regulation of PKA signaling, were also affected by consumption of an HFD (Fig. 2d). Analysis of hypothalamic samples from mice fed either ND or HFD uncovered a decrease in the total phosphorylation levels of PKA substrates in samples from HFD-fed animals (Fig. 2e) and decreased phosphorylation at serine 133 of CREB, a key downstream target of PKA (Fig. 2f).

**Table 1:** Changes in expression of genes regulating the PKA signaling pathway following HFD

Entrez gene name	Fold change	*P*-value	False discovery rate (*q*-value)
Adenylate cyclase 1 (brain)	−1.605	9.77E–07	5.09E–03
Lymphoid enhancer-binding factor 1	−2.577	2.72E–06	9.62E–03
Myosin, light chain 2, regulatory, cardiac, slow	−2.137	4.51E–05	2.80E–02
Protein kinase C, delta	−5.952	1.30E–05	1.89E–02
Protein tyrosine kinase 2 beta	−1.634	1.29E–05	1.89E–02
Protein tyrosine phosphatase, non-receptor type 3	−5.155	5.65E–05	3.07E–02
Protein tyrosine phosphatase, non-receptor type 4 (megakaryocyte)	12.114	1.88E–07	4.25E–03
Transcription factor 7-like 2 (T-cell specific, HMG-box)	−2.703	5.60E–05	3.07E–02

These results suggest that the consumption of a HFD regulates the PKA signaling pathway in the hypothalamus and might be responsible for the development of the obesity-induced depression-like phenotype in mice.

### HFD increases expression levels and activity of PDE4A5 in the hypothalamus

Next, we sought to investigate whether DIO alters the activity of PDE4 enzymes in the hypothalamus. There was a slight trend, but one that did not reach statistical significance, for increased total PDE4 activity in mice fed an HFD for 3 weeks vs. mice fed an ND (Supplementary Fig. S3A).

**Fig. 3:**
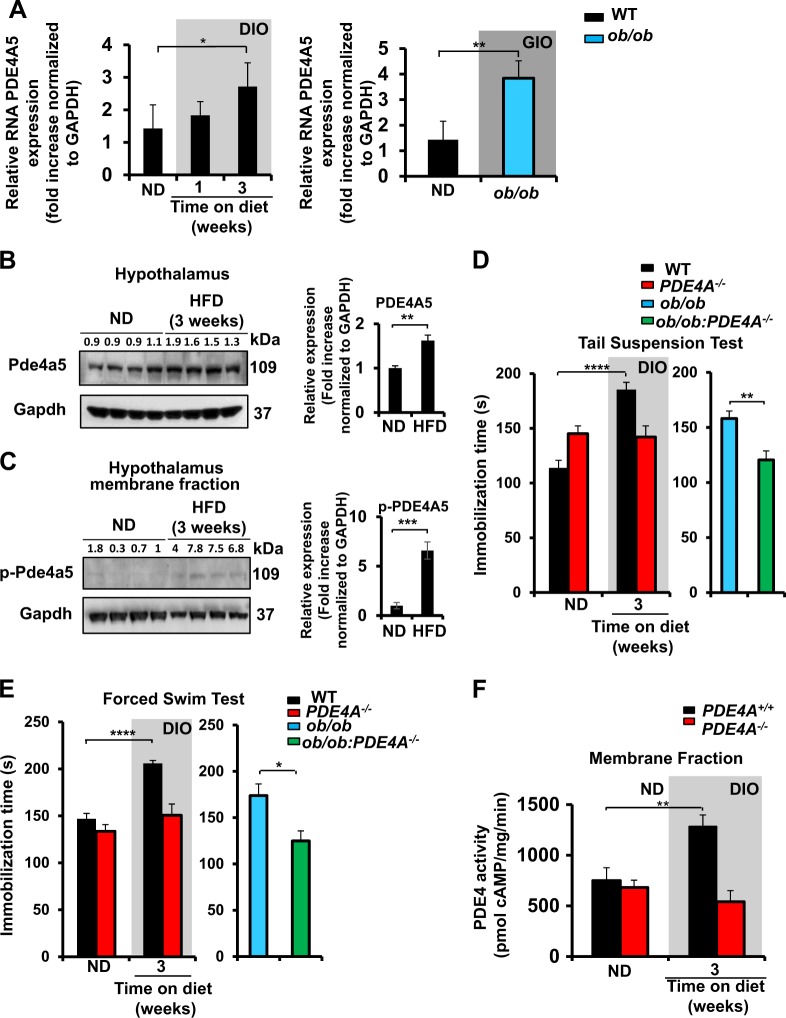
PDE4A is involved in the depression-like phenotype induced by obesity and HFD increases expression levels and activity of phosphodiesterases 4A5 in the hypothalamus. **a** Real-time PCR analysis of *PDE4A5* mRNA in the hypothalamus of (left) WT C57BL/6J mice fed either ND or HFD (1 and 3 weeks) (*n* = 4 per condition; *P < 0.05, by one-way ANOVA with Dunnett’s multiple comparison test) and (right) between WT C57BL/6J mice and *ob/ob* mice (*n* = 4 per condition; **P < 0.01 by two-tail unpaired Student's *t*-test). **b** (Left) Western blot analysis of Pde4a5 expression in WT C57BL/6J mice fed either ND or HFD. (*n* = 4 mice per condition, **P < 0.01 by two-tail unpaired Student's *t*-test). Representative blot (left) and (right) its quantification. **c** Western blot analysis of phosphorylated-Pde4a5 (p-Pde4a5) expression in WT C57BL/6J mice fed either ND or HFD. (*n* = 4 mice per condition, ***P < 0.001 by two-tail unpaired Student's *t*-test). Representative blot (left) and its quantification (right). Gapdh was used as the loading control for all western blots shown in this figure. All data in the figure are represented as mean ± SEM. **d** Tail suspension and **e** forced swim tests on *PDE4A*^*+/+*^ and *PDE4A*^*−/−*^ mice fed either normal diet (ND) or high-fat diet (HFD) (*n* = 8–10 mice per condition; ****P < 0.0001 by two-way ANOVA with Sidak’s multiple comparison test) and *ob/ob* vs *ob/ob*::*PDE4A*^*−/−*^ mice fed ND (*n* = 7–10 mice per condition; **P < 0.01, *P < 0.05 by two-tail unpaired Student's *t*-test) DIO: diet-induced obesity. **f** PDE4 activity in the membrane fraction of hypothalamus collected from *PDE4A*^*+/+*^ and *PDE4A*^*−/−*^ mice fed either ND or HFD (*n* = 5–6 mice per group; **P < 0.01 by two-way ANOVA). All data in the figure are represented as mean ± SEM

A variety of different *PDE4* isoforms are expressed in the brain, so we decided to perform real-time PCR analysis to investigate whether DIO or GIO in mice can alter the mRNA levels of particular *PDE4* isoforms in the hypothalamus. Levels of *PDE4B* mRNA in the hypothalamus were undetectable, whereas no statistically significant difference was found for *PDE4D* transcripts among mice fed ND, mice fed HFD or *ob/ob* mice (Supplementary Fig. S3B). In contrast to this, the total levels of the *PDE4A* isoforms were somewhat increased, in response to DIO and GIO, although such changes did not attain statistical significance (Supplementary Fig. S3C). However, when we analyzed transcript levels for each of the different *PDE4A* isoforms encoded by the *PDE4A* gene, then we found that transcripts for the PDE4A5 isoform were specifically upregulated in response to both DIO and GIO (Fig. 3a). Furthermore, PDE4A5 protein levels were increased in the hypothalamus after 3 weeks on the HFD (Fig. 3b), as was the level of PKA-mediated phosphorylation of the PDE4A5 population that was located within the membrane fraction (Fig. 3c). PKA phosphorylation of PDE4 long isoforms, such as PDE4A5, has been shown to elicit their activation, which serves as a critical negative feedback loop by engendering increased cAMP degradation^37^.

Such results of ours revealed that both DIO and GIO lead to the specific upregulation of the PDE4A5 isoform in the hypothalamus. Furthermore, the level of protein expression and the PKA phosphorylation-mediated activation status of PDE4A5 were both increased in the hypothalamus of HFD-fed mice.

### PDE4A is involved in the depression-like phenotype induced by obesity

Given the potential central role of PDE4A, we assessed whether mice lacking *PDE4A* (*PDE4A*^*−/−*^) were protected from the depression-like behavior induced by obesity. Genetic ablation of *PDE4A* in vivo prevented both DIO and GIO depression, as shown by the tail suspension and forced swim tests (Fig. 3d, e). *PDE4A*^*−/−*^ and their WT litter mate controls (*PDE4A*^*+/+*^) showed similar increases in body weight when maintained on ND or HFD (Supplementary Fig. S3D, S3E). The *ob/ob* and the double knockout *PDE4A*^*−/−*^:*ob/ob* showed similar body weight gains when fed an ND (Supplementary Fig. S3E). These results suggest that loss of PDE4A protects mice from obesity-associated depression phenotype, despite similar weight gains in response to an HFD.

To elucidate further any subcellular regulation of PDE4, due to the consumption of HFD, PDE4 activity assays were performed on both cytosolic and membrane fractions from hypothalamus. PDE4 activity was greater in the membrane fraction of mice fed an HFD for 3 weeks than on an ND (Fig. 3f). This increase was abolished in the *PDE4A*^*−/−*^ mice (Fig. 3f), suggesting that membrane-associated PDE4A, namely PDE4A5, is the functionally relevant PDE4A species whose activity is upregulated in the hypothalamus after the consumption of a HFD for 3 weeks. No difference was detected for PDE4 activity in the cytosolic fraction of either WT or *PDE4A*^*−/−*^ mice maintained on ND or HFD (Supplementary Fig. S3F).

The amygdala is involved in depression with many neuronal circuits within the hypothalamus; however, this brain region showed no statistical difference in PDE4 activity levels between the ND and HFD in either WT or *PDE4A*^*−/−*^ mice (Supplementary Fig. S4A, S4B). Other brain areas involved in the depression-related behaviors, such as the cortex, hippocampus, and cerebellum, also showed no differences in PDE4 activity differences among mice fed either an ND or HFD (Supplementary Fig. S4C-E), further suggesting that the hypothalamus is a key locus affected in obesity-induced depression that leads to the upregulation of PDE4 activity.

The behavior phenotype induced by the GIO and DIO was not due to the development of any motor or anxiety deficits (Supplementary Fig. S5A, S5B). Measurement of motor anxiety (open field test) revealed no major differences between WT and *PDE4A*^*−/−*^ fed either ND or HFD or between the *ob/ob* and *PDE4A*^*−/−*^:*ob/ob mice* (Fig. S5A). Use of the elevated plus maze test for the anxiety phenotype showed no difference between WT and *PDE4A*^*−/−*^ mice or between the *ob/ob* and *PDE4A*^*−/−*^:*ob/ob* (Supplementary Fig. S5B).

The loss of *PDE4A* gene products prevented both the DIO and GIO in mice. Moreover, from all the brain regions that have been shown to be involved in the neurocircuitry of depression hypothalamus is the specific brain region with increased PDE4 activity due to the development of DIO. Furthermore, we found that the membrane compartmentalization of cAMP hydrolyzing PDE4A activity is critical for the cAMP/PKA signaling pathway in the depression-like phenotype.

### Saturated fats accumulate specifically in the hypothalamus of mice fed the HFD and they can regulate PKA signaling in a neuronal cell line

**Fig. 4:**
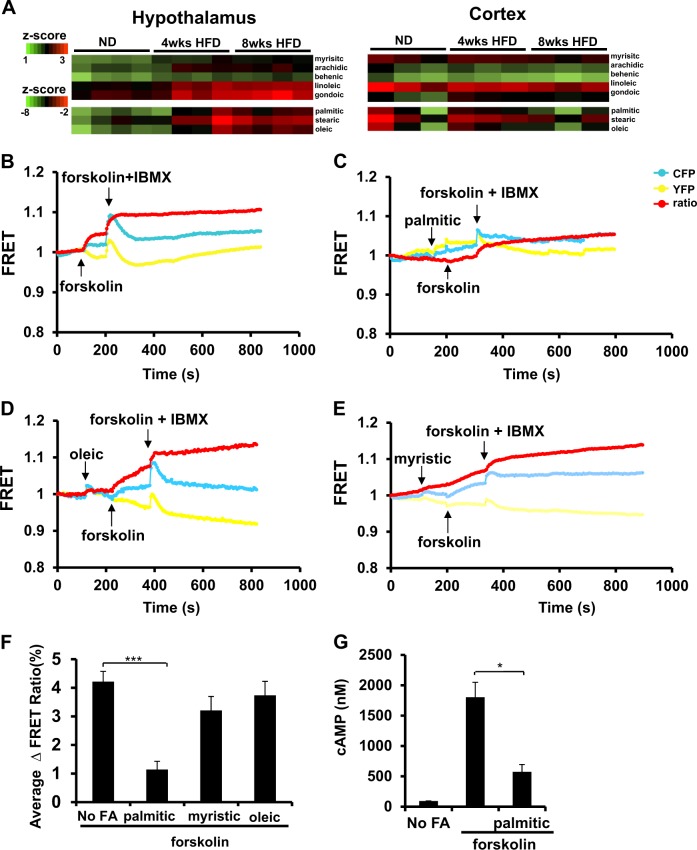
Saturated fats accumulate specifically in the hypothalamus of mice fed the HFD and they can regulate PKA signaling in a neuronal cell line. **a** Fatty acid profile (heatmaps) for hypothalamic (left) and cortical (right) samples collected from wild-type (WT) C57BL/6J mice fed either a normal diet (ND) or a high-fat diet (HFD) for a period of 4 or 8 weeks. Heatmaps were divided in two groups of high or low representation of fatty acids in the brain. Colors represent values as log_10_ after normalization by z-score. Low expression level is shown in green and high expression level in red. (*n* = 3–4 mice per condition analyzed by two-way ANOVA with Bonferroni post-hoc test). **b** Mouse neuroblastoma cell line (N2a) transfected with rat PDE4A5-wt and PKA-R1 Förster (or fluorescence) resonance energy transfer (FRET) and treated with forskolin alone, **c** 100 μM palmitic acid before forskolin stimulation, **d** 100 μM oleic acid before forskolin stimulation, or **e** 100 μM myristic acid before forskolin stimulation. At the end of each experiment, cells were treated with the generic PDE inhibitor 3-isobutyl-1-methylxanthine (IBMX) to test for their responsiveness. **f** Quantification of the PKA activation by resonance energy transfer in N2a cells pretreated with 100 μM of palmitic, oleic, or myristic acids. (*n* = 6–8 individual N2a cells per condition, ***P < 0.001 by one-way ANOVA with Dunnett’s multiple comparison test). **g** Measurement of cAMP in N2a cells treated with forskolin alone or in combination with palmitic acid (*n* = 3 independent replicas per condition; *P < 0.05, by unpaired two-tail Student's *t*-test. Two independent experiments). All data in the figure are represented as mean ± SEM

Next, we hypothesized that dietary fatty acids might play pivotal roles as molecular transducers of cell signaling in the hypothalamus to regulate mood disorders such as depression. Increased accumulation of FFAs in the hypothalamus was found among mice fed an HFD for either 4 or 8 weeks on HFD compared with mice fed an ND (Fig. 4a, Supplementary S6A). One of the fatty acids with the highest upregulation in the hypothalamic samples of mice fed an HFD compared with mice maintained on the ND was the palmitic acid (Fig. 4a, Supplementary S6A). By contrast, fatty acid profile analysis of the cortex revealed no differences between the two dietary groups (Fig. 4a), suggesting that the hypothalamus was a specific brain region with FFAs accumulation after HFD.

To test whether dietary fatty acids have a direct role in regulating PKA signaling in a neuronal cell line, we used a Förster (or fluorescence) resonance energy transfer (FRET)-based biosensor, based on the structure of PKA, to gauge dynamic cAMP signaling^38^. This probe enables quantitative, real-time detection of rapid changes in cytosolic PKA activity after cell treatment. Treatment of a neuronal cell line, which had been co-transfected to express both PDE4A5 and the PKA-R1 FRET sensor, with forskolin, an adenylyl cyclase activator, led to a marked increase in cellular PKA activity (Fig. 4b, f). However, pretreatment of such cells with palmitic acid abolished the forskolin-induced PKA activation (Fig. 4c, f). This effect was specific to palmitic acid as neither oleic acid nor myristic acid had any effect on forskolin-induced PKA activation in such cells (Fig. 4d–f). In accordance with this, pretreatment of the cells with palmitic acid abolished the forskolin-induced increase in cAMP levels in such cells (Fig. 4g).

These data indicate a differential effect of fatty acids on intracellular PKA activity. Namely, consumption of an HFD leads to an efflux of dietary fatty acids specifically in the hypothalamus and the entrance of dietary palmitic acid in the brain suppress the PKA pathway. Moreover, different fatty acids have differential effects on the PKA signaling cascade in a neuronal cell line.

### The increased accumulation of the dietary fatty acid palmitic correlates with the upregulation of the *FFAR1* and modulates the association of this receptor with PDE4A5

**Fig. 5:**
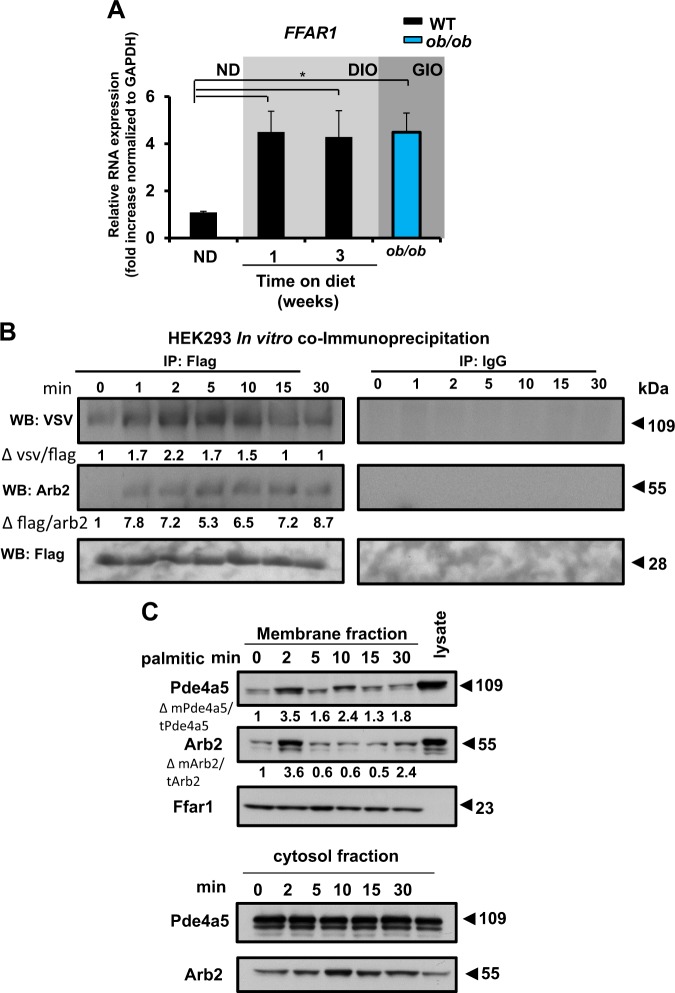
The increased accumulation of the dietary fatty acid palmitic correlates with the upregulation of the *FFAR1* and modulates the interaction of this receptor with Pde4a5. **a** Real-time PCR analysis of *FFAR1* mRNA in the hypothalamus of wild-type (WT) C57BL/6J mice fed a normal diet (ND), WT C57BL/6J mice fed a high-fat diet (HFD) for 1 week and 3 weeks, and *ob/ob* mice (*n* = 4–9 mice per condition. Experiment was repeated three times, *P < 0.05, by one-way ANOVA with Bonferroni multiple comparison test). Data are represented as mean ± SEM. **b** Co-immunoprecipitation of Pde4a5 with Ffar1 in a human embryonic kidney cell line (HEK293) treated with 500 μM of palmitic acid at various time points. Pde4a5 was tagged with VSV (Vesicular stomatitis virus). β-Arrestin-2 (Arb2) was tagged with Flag. VSV tag and Arb2 levels normalized to Flag tag were quantified by densitometry (Δ represents fold changes). Similar results from two independent experiments were obtained and a representative immunoblot is shown. **c** Membrane fractionation of N2a cells treated with 500 μM of palmitic acid at various time points. Ffar1 was used to identify the membrane fraction. Pde4a5 and Arb2 levels normalized to total levels and quantified by densitometry (Δ represents fold changes). Similar results from two independent experiments were obtained and a representative immunoblot is shown

We next sought to investigate whether the expression of the different FFA receptors is altered in the hypothalamus of mice upon DIO or GIO. Real-time PCR analysis revealed a statistically significant upregulation of *FFAR1* in the hypothalamus in response to an HFD and in the *ob/ob* mouse (Fig. 5a). Hypothalamic gene expression of *FFAR3*, a receptor that belongs in the same family as *FFAR1*, was not affected after the consumption of the HFD (Supplementary Fig. S6B); however, the *FFAR4* receptor was increased in the hypothalamus of the *ob/ob* mouse (Supplementary Fig. S6C).

Next, we tested whether the PDE4A5 isoform interacts with Ffar1. In vitro co-immunoprecipitation assays from lysates derived from a human embryonic kidney cell line (HEK293) exhibited a time-dependent interaction between Ffar1 and PDE4A5 after treatment with palmitic acid (Fig. 5b). Palmitic acid treatment increased the translocation of the PDE4A5 protein, as well as β-arrestin-2, to the membrane fraction of the N2a cells in a time-dependent manner (Fig. 5c). Given that PDE4A5 is known to bind to β-arrestin-2^39^, these data are consistent with the translocation of a PDE4A5–β-arrestin-2 complex to the membrane (Fig. 5b, c). Oleic acid treatment did not induce the translocation of PDE4A5 to the membrane, highlighting the specificity of the fatty acid receptor–ligand signaling cascade (Supplementary Fig. S6D).

These data suggest that HFD specifically increases *FFAR1* gene expression in the mouse hypothalamus, which in turn can lead to a potential association of Ffar1 and PDE4A5. The translocation of PDE4A5 to the membrane fraction will lead to a re-programming of the pattern of compartmentalization of the cAMP signaling pathway in these cells.

## Discussion

Using behavioral paradigms in mice, we demonstrated, as have others previously^40,41^, that either DIO or GIO can be causative for the development of depression. This relationship is mechanistically coupled to regulation of cAMP/PKA signaling in the hypothalamus. Interestingly, such an effect is independent of the increases in body weight caused by consumption of an HFD or induction of stress as shown by the EPM behavioral assay.

Protein and mRNA analysis identified PKA signaling as the main pathway altered in the hypothalamus after consumption of an HFD. PKA is a tetrameric enzyme that phosphorylates its protein targets when cAMP binds its regulatory subunits^42^. The PKA signaling cascade and depression have been previously linked^14^—but not in the context of diet composition—as chronic administration of antidepressant drugs or electroconvulsive seizures targets PKA signaling in the brain^16,43^. The present study reveals that the accumulation of different fatty acids in the hypothalamus alters PKA signaling, suggesting a potential mechanism of action of dietary fatty acids in the regulation of mood disorders, such as depression, via the PKA signaling pathway. Although mice show depressive behavior after 3 weeks on HFD, most of the PKA-mediated gene expression manifest at 8 weeks. This can be explained by the fact that transient changes in cAMP can lead to both short and delayed/extended gene expression changes. For example, short- and long-term memory actions^44^. Another potential mechanism that may result in the development of obesity-induced depression phenotype is inflammation^45^. Indeed, in this regard, palmitic acid has been shown to activate Toll-like receptor 4 (TLR4) signaling^46^.

To the best of our knowledge, the present findings are the first to show that the consumption of an HFD induces an influx of dietary fatty acids specifically in the hypothalamus, leading to an impairment of the cAMP/PKA signaling cascade and this downregulation of the PKA pathway can be implicated behaviorally for the development of depression in mice.

Signaling via cAMP is downregulated among patients with depression^47^. Many antidepressant drugs act by upregulating molecules involved in cAMP signaling, which is the major regulator of PKA^15^. Cyclic nucleotide PDEs provide the sole route for cellular degradation of cAMP^48^, with each PDE isoform displaying distinct roles and intracellular localization^26^. PDE4 enzymes are major regulators of the cAMP signaling in the brain and localize in brain regions that are associated with reinforcement, movement, and affect, all of which actions are altered among people with depression^49^. A similar mechanism of the action of antidepressant drugs that act by the upregulation of the cAMP signaling pathway has been proposed for rolipram, a selective PDE4 inhibitor with known antidepressant activity in mice^50^. Chronic administration of rolipram leads to a sustained elevation of cAMP levels^51^ and increases the expression of CREB, brain-derived neurotrophic factor (BDNF) and tropomyosin receptor kinase B (TrkB), all of which are believed to facilitate the action of antidepressants^18,52^. Despite initial promise, the therapeutic potential of rolipram as an antidepressant has been limited by compromising adverse side effects, particularly nausea and vomiting^50,53^ because this compound inhibits all PDE4 isoforms^54^.

Identifying the specific PDE4 isoform that mediates the antidepressant action of rolipram could enable the development of selective inhibitors that offer therapeutic effects with minimal adverse reactions^55^. Here we show that the loss of *PDE4A* in vivo prevented the depression-like phenotype observed in mice in response to DIO or GIO. PDE4A5 appears to be the specific PDE4 isoform responsible for the depression phenotype. Consumption of an HFD increases the PDE4 activity specifically in the hypothalamus. Of note, such an increase was abolished in the PDE4A^*−/−*^ mouse model. Levels of PDE4A5 mRNA and protein (including the phosphorylated form) were higher in hypothalamic samples collected from mice fed an HFD versus an ND. Interestingly, it has been previously shown that PDE4A5 interacts with disrupted in schizophrenia 1 (DISC1), a major genetic risk factor for the development of schizophrenia^56^. Therefore, our novel findings suggest that PDE4A5 may have potential therapeutic importance for the design of a PDE4A5, isoform-selective inhibitor that would minimize the adverse effects associated with the use of a generic PDE4 inhibitor (note that the cognate enzyme in humans is termed PDE4A4). Such a novel, isoform-selective inhibitor might rescue the depression phenotype caused by obesity.

Considerable focus has been placed on developing agents targeting monoamines and their metabolism^8^ for the treatment of depression. However, 50% of all patients do not respond to the currently available antidepressant drugs^57^. Moreover, the majority of overweight and obese individuals do not respond to current antidepressant treatments, which suggested that other molecular pathways are involved in the development of depression among this subpopulation^10^. Interestingly, a previous connection between activation of PDE4A isoforms by fatty acids has been established in immune cells^58,59^. FFA receptors in the brain might explain how dietary fatty acids can link food intake with mood disorders such as depression. Regulation of the expression of different FFA receptors at the mRNA level, especially FFAR1, in the hypothalamus in response to DIO and GIO represent a potential mechanism to regulate depression. Despite the potential role of FFAR1 signaling in the hypothalamus for lipid sensing that controls energy balance and food intake^13^, the present study shows for the first time that FFAR1 signaling might also play an important role in mood disorders such as depression. There was a trend for the FFAR3 to increase with the consumption of an HFD, however, it did not reach statistical significance. This might be due to the small number of animals used for the real-time PCR analysis. Further studies, however, are needed to characterize any potential involvement of the short chain fatty acid receptor FFAR3 in contributing to the phenomenon we uncover here, namely of a novel, obesity-induced depression phenotype. As such, in addition to the established role of fatty acid receptors predominantly acting in the regulation of metabolic pathways, such as insulin secretion^60^, data in this study suggest that fatty acid receptors in the brain may promote signaling related to mood disorders.

In conclusion, our study shows that FFAR1 associates with the PDE4A5 isoform. This discovery highlights the possibility that developing small molecules aimed at inhibiting the association between PDE4A5 and FFAR1 could provide novel therapeutics for treating patient’s depression caused by their diet. Further studies are required, however, to investigate the potential for either a direct interaction of FFAR1 and PDE4A5 or an indirect one involving β-arrestin. Determination of the exact interaction sites for these species is needed to better understand that pathway and to develop novel therapeutics based upon disrupting the interaction of such components. Indeed, small molecules that selectively target the interaction of the PDEs with FFA receptors might represent a new generation of antidepressants with increased specificity for either overweight and/or obese individuals.

## Materials and methods

### Mice and diets

WT C57BL/6J mice and leptin-deficient mice (Lep^ob^ or *ob/ob*) on a C57BL/6J background were obtained from The Jackson Laboratory. The *PDE4A*^*−/−*^ mouse line was a kind gift from Marco Conti that was generated as described^61^ and crossed with a C57BL/6J background (11 or 12 crossings). Heterozygous *PDE4A*^*+/−*^ were crossed to obtain *PDE4A*^*−/−*^ and *PDE4A*^*+/+*^ male littermates that were used in this study and had access to food and water ad libitum. All animal study protocols and procedures were reviewed and conducted in accord with the Guide for the care and use of laboratory animals (LARC) at UCSF, approved by the institutional animal care and use committees of UCSF, and are in compliance with standards set by the National Institutes of Health. Mice were fed a ND (LabDiet 5053) or a HFD (Research Diets 12492) for 3 or 8 weeks.

### Behavioral assays

Behavioral tests were conducted to assess the depression phenotype to the following sequence: open field, elevated plus maze, sucrose preference test, tail suspension, and forced swim test. Open field test was used to assess the total locomotor activity using the Digiscan locomotor activity monitor (Model RXYZCM, Omnitech Electronics; Columbus, OH). Tail suspension^62^ and forced swim^63^ tests were performed to assess the depression-related phenotype. In both tests, immobilization time is defined as the average time the mouse does not struggle to escape in these tests. A modified protocol of the sucrose preference test^64^ was performed to assess the anhedonia phenotype that correlates with depression in humans. Briefly, for 3 days prior to experimental start, mice were singly housed and habituated with ad libitum food and drink from two bottles: tap water and a 2% sucrose solution. Bottles were reversed every day throughout the time of the habituation to avoid side preference. On the day of the test, mice were deprived from both water and sucrose for 8 h. At the end of the day, the two bottles were put back for 2 h. Bottles were placed in a different order in every cage to avoid side preference. The consumption of water and sucrose solution was estimated simultaneously in control and experimental groups by weighing the bottles. The preference for sucrose was calculated as a percentage of total liquid consumed. The elevated plus maze test was performed for the measurement of anxiolytic or anxiogenic behaviors in rodents^65^.

### Gene expression analysis

RNA extraction, reverse transcription, and real-time PCR were performed as described^66^. Primers used were: PDE4A: Fwd 5′-CGAGCACTACAGTGGTGGAA-3′, Rev 5′-AAAAGGATCAGGCAGGGTCT-3′; PDE4B: Fwd 5′-GTCCCAGGTTGGTTTCATTG-3′, Rev 5′-ACACAGGGATGGAATCGAAG-3′; PDE4D: Fwd 5′-GTCCCATGTGTGACAAGCAC-3′, Rev 5′-TCAGTGTCTGACTCGCCATC-3′; PDE4A5: Fwd 5′-TCGCCGCACCGGCCCATAGA-3′, Rev 5′-GACGAGGGCCAGGACATGCG-3′; FFAR1: Fwd 5′-AATGCCTCCAATGTGGCTAG-3′, Rev 5′-AGTCCTCGTCACACATATTG-3′, FFAR3: Fwd 5′-CTTGTATCGACCCCCTGGTTTT-3′, Rev 5′-GCTGAGTCCAAGGCACACAAGT-3′; FFAR4: Fwd 5′-TTCATATGGGGTTACTCGGC-3′, Rev 5′-GATTTCTCCTATGCGGTTGG-3′; GAPDH: Fwd 5′-CAAGGCCGAGAATGGGAAG-3′, Rev 5′-GGCCTCACCCCATTTGATGT-3′.

### Gene expression profiling by microarray analysis

Microarray analysis was performed on hypothalamic areas of mice fed a ND or a HFD for 4 or 8 weeks as described^66^. Briefly, hypothalamic area was dissected using the brain slicer matrix (Zivic Instruments), and total RNA was isolated with RNeasy Mini kit/RNeasy Lipid tissue mini kit (Qiagen). Probes were prepared using NuGEN Ovation Pico WTA V2 kit and NuGEN Encore Biotin Module, and hybridized to Rat and Mouse Gene 1.0 ST GeneChip arrays (Affymetrix). Arrays were scanned using an Affymetrix GCS3000 scanner and Affymetrix Command Console software, and data were normalized using the RMA algorithm in Affymetrix Expression Console. Microarrays were normalized for array-specific effects using Affymetrix’s “Robust Multi-Array” (RMA) normalization and were reported on a log_2_ scale. For statistical analyses, we removed all array probe sets in which no experimental groups had an average of log_2_ intensity >3.0. Linear models were fit for each gene using the Bioconductor “limma” package in R^67^. Moderated *t-*statistics, fold change, and the associated *P*-values were calculated for each gene. To account for the fact that thousands of genes were tested, we reported FDR-adjusted values, calculated using the Benjamini–Hochberg method^68^. Pathway analysis was performed using the GO enrichment and Ingenuity IPA analysis.

### Subcellular fractionation, western blotting, and antibodies

Tissue or cell extracts were lysed in TNE buffer (10 mM Tris pH 8.0, 150 mM NaCl, 1 mM EDTA, 1% NP40) supplemented with protease and phosphatase inhibitors (Calbiochem). Lysates were centrifuged at 4 °C at 16,000 × *g* for 15 min and the supernatant was stored at −80 °C for further protein analysis. For the membrane–cytosol fractionation experiments, cells or tissue were lysed in HKEM buffer (50 mM KCl, 50 mM HEPES, KOH pH 7.2, 10 mM EGTA, 1.92 mM MgCl_2_) supplemented with protease and phosphatase inhibitors (Calbiochem) and placed on ice for 30 min. Lysates were spun down for 10 min at 1000 *g* at 4 °C, and the supernatant was centrifuged at 100,000 *g* for 1 h at 4 °C. The supernatant was saved as the cytosol fraction. The pellet was resuspended in KHEM buffer + 1% Triton and 150 mM NaCl and incubated for 30 min on ice with occasional agitation before the second centrifugation for 100,000 *g* for 1 h at 4 °C. The supernatant was saved as the membrane fraction. Equal amounts of protein were then analyzed by western blotting as described^69^. The following antibodies were used: rabbit anti-Gapdh (1:4000; Abcam), rabbit anti-Creb (1:1000; Cell Signaling), rabbit anti-phospho-Creb (1:2000; Cell Signaling), rabbit anti-Ffar1 (1:2000; Abcam), mouse anti-βarrestin-2 (1:800; Santa Cruz), mouse anti-Flag (1:3000; Sigma), rabbit anti-phospho-Pka (1:1000; Cell Signaling), and mouse anti-Vsv (1:4000; Sigma). For the detection of PDE4A and phospho-PDE4A, antibodies raised in rabbit and produced in house were used at 1:1000 as described^70^. Densities of the protein bands were measured by ImageJ software, and the statistical analysis was done by Graphpad prism 7.

### Cell culture and in vitro fatty acid treatment and co-immunoprecipitation (Co-IP)

The human embryonic kidney 293 (HEK293) cells were maintained in growth medium containing Dulbecco’s modified Eagle’s medium (DMEM) supplemented with 10% (v/v) fetal bovine serum, 1% (v/v) l-glutamine, and 1% (v/v) penicillin–streptomycin. The mouse neuroblastoma cell line (N2a) were maintained in growth media containing DMEM supplemented with 10% (v/v) fetal bovine serum, 1% (v/v) l-glutamine, and 1% (v/v) penicillin–streptomycin and 1% (v/v) non-essential amino acid. Transfections were performed at 50–60% confluence with 5 μg of total circular plasmid DNA using the linear MW~25000 polyethylenimine (Polysciences Inc.). For the HEK293, we used 2 μg of PDE4A5-vsv with 2 μg of GPR40 and 1 μg of arrestin-2. For the N2a, we used 2.5 μg of PDE4A5-vsv with 2.5 μg of arrestin-2. After 24-h transfection, the medium was replaced with fresh pre-warmed culture medium and further incubated for 24 h before the cells were treated with palmitic, oleic, and myristic (Nu-check). Treatment of cells with the different fatty acids was done by reducing the serum before the actual experiment. Co-IP was performed as described^71^. Briefly, cell lysates were prepared in 1% Nonidet P-40, 150 mM NaCl, 1 mM EDTA, and 10 mM Tris·HCl, pH 8.0 supplemented with phosphatase and protease inhibitors. Immunoprecipitations (IPs) were performed with an anti-Flag antibody and immunoblot with anti-Vsv and Arrestin-2β (ARB2).

### PDE4 activity assays

The PDE4 activity in the brain homogenates was assayed as described^72^. The samples were lysated in KHEM buffer (50 mM KCl, 50 mM HEPES pH 7.2, 10 mM EGTA, 1.9 mM MgCl_2_) supplemented with protease (COmplete EDTA-free, Roche) and phosphatase (PhosSTOP, Roche) inhibitor cocktail tablets. Pilot assays were carried out to verify PDE4 activity and ensure activity fell within the linear range of 6000–16,000 counts. Each sample was done in triplicate and was incubated with and without the PDE inhibitor rolipram. Rolipram was dissolved in 100% dimethyl sulfoxide (DMSO) as a 10 mM stock solution and diluted in 20 mM Tris/HCl, 10 mM MgCl_2_ buffer (final pH 7.4) to a final concentration of 10 μM. The difference between the two different measurements represents the specific PDE4 activity in each sample.

### cAMP measurement of N2a cells

Measurements of cAMP were performed using the CatchPoint Cyclic-AMP fluorescent assay kit (Molecular Device, CA). Briefly, cells were lysed in lysis buffer provided by the manufacturer and left on ice for 15 min. Lysates were spun down for 10 min at 1000 *g* at 4 °C (low-speed pellet, cell debris, and nuclei) and the supernatant assayed according to the manufacturer’s instructions.

### Fatty acid analysis by gas chromatography–mass spectrometer

The total concentrations of palmitic acid (16:0), stearic acid (18:0), myristic acid (14:0), behenic acid (22:0), arachidic acid (20:0), gondoic (20:1), oleic (18:1), and linoleic (18:2) were determined from tissues by gas chromatography–mass spectrometry^73^. A known quantity of tissue was hydrolyzed and extracted after adding a known amount of heptadecanoic acid (17:0). Fatty acids were analyzed as their trimethylsilyl derivatives under electron impact ionization mode using an Agilent 5973N-MSD equipped with an Agilent 6890 GC system and a DB17-MS capillary column (30 m × 0.25-mm internal diameter × 0.25-μm film thickness).

### FRET imaging

FRET imaging experiments were performed 24–48 h after transfection with the PKARI sensor on mouse neuroblastoma (N2a) cell line that were seeded onto glass cover slips. The PKARI probe is based on the AKAP-binding domain of PKA-RIa fused to the cAMP-binding domain of EPAC. Cells were maintained at room temperature in dulbecco’s phosphate buffered saline (DPBS) (Invitrogen, UK), with added CaCl_2_ and MgCl_2_, and imaged on an inverted microscope (Olympus IX71) with a PlanApoN, 60 × , NA 1.42 oil, 0.17/FN 26.5, objective (Japan). The microscope was equipped with a CCD camera (cool SNAP HQ monochrome, Photometrics), and a beam-splitter optical device (Dual-channel simultaneous-imaging system, DV^2^ mag biosystem (ET-04-EM)). Imaging acquisition and analysis software used was Meta imaging series 7.1, Metafluor, and processed using ImageJ (http://rsb.info.nih.gov.ucsf.idm.oclc.org/ij/). FRET changes were measured as changes in the background-subtracted 480/545-nm fluorescence emission intensity on excitation at 430 nm and expressed as either *R*/*R*0, where *R* is the ratio at time *t* and *R*0 is the ratio at time = 0 s, or Δ*R*/*R*0, where Δ*R* = *R*–*R*0. Values are expressed as the mean ± SEM. Cells were pretreated with 100 μM of either palmitic or oleic before 5 μM of forskolin treatment. At the end of every experiment, saturated doses of forskolin (25 μM) or IBMX (100 μM) were used to check for the responsiveness of the cells.

### Statistical analysis

Data were expressed as mean value ± standard error of the mean (SEM) and an alpha level of 0.05 was used as marker of statistical significance. Statistical significances between two groups of data were determined using unpaired, two-tailed Student’s *t*-test. Statistical analysis of several groups was carried out either by using two-way analysis of variance (ANOVA) with different post-test comparisons against control experiments using GraphPad Prism 7 or mixed model analysis with fixed and random factors using R. A *P*-value >0.05 was not considered significant (NS), *P*-value < 0.05 was labeled as (*), *P*-value < 0.01 was labeled as (**), and *P*-value < 0.001 was labeled as (***).

## Supplementary information

Supplemental figure legends

Supplementary Figure 1

Supplementary Figure 2

Supplementary Figure 3

Supplementary Figure 4

Supplementary Figure 5

Supplementary Figure 6
