# Supplementary material for: A high-fat diet promotes depression-like behavior in mice by suppressing hypothalamic PKA signaling
Source: Transl Psychiatry. 2019 May 10;9:141. doi: 10.1038/s41398-019-0470-1 (PMC6510753; doi:10.1038/s41398-019-0470-1)
Supplement: Supplementary file 1 — Supplemental figure legends [file 41398_2019_470_MOESM1_ESM.docx]

**Supplemental Information**

**Figure S1. Dietary or genetically induced obesity induces depression-like behavior in mice.** (**A**) SPT for WT C57BL/6J mice maintained either on ND or on HFD for a period of 3 or 8 weeks. (n=5–8 mice per group, experiment was performed once; ***P* < 0.01 by linear mixed-model fit by REML. (**B**) Comparison of the increase in body weight of WT C57BL/6J mice on HFD for 10 weeks and their litter mate age-matched mice on ND (n=10 per group, experiment repeated twice; ***P* < 0.01 by linear mixed-model fit by (REML). All data in the figure are represented as mean ± SEM. See also Fig. S.1. (**C**) Correlation graphs between immobilization time and body weight for wild type (WT) C57BL/6J mice fed a normal diet (ND) versus mice fed a high-fat diet (HFD) for 3 weeks. Data are shown for the tail suspension test and the forced swim test with their respective r^2^ and p values (n=25–29 mice per group).

**Figure S2. The consumption of a HFD does not affect total activity levels.** (**A**) Open field test for WT C57BL/6J mice fed ND, WT C57BL/6J mice fed HFD (3 and 8 weeks), and *ob/ob* mice fed ND (12-16 weeks). Measurements are given for total activity in the entire or central area of the open field apparatus, as well as rearing (n=7–10 mice per condition). The WT and *ob/ob* mice were compared (**P<0.01, ****P<0.0001, one-way ANOVA with Tukey’s multiple comparison test). All data in the figure are represented as mean ± SEM. (**B**) Comparison of the increase in the body weight of the *ob/ob* mice versus the WT C57BL/6J aged-matched controls (n=6–11 per group, experiment was performed once ****P* < 0.001 by linear mixed-model fit by restricted maximum likelihood (REML). All data in the figure are represented as mean ± SEM.

**Figure S3. The loss of *PDE4A* does not affect body weight either on ND or HFD.** (**A**) Phosphodiesterase 4 (PDE4) activity in whole-hypothalamic homogenates of wild type (WT) C57BL/6J mice fed either a normal diet (ND) or DIO for 3 weeks (n=7–10 mice per condition).

(**B**) Real-time PCR analysis of *PDE4D* mRNA and (**C**) *PDE4A* mRNA in the hypothalamus of WT C57BL/6J mice fed ND, WT C57BL/6J mice fed HFD for 1 week or 3 weeks and *ob/ob* mice (n=4 mice per condition). **(D)** Body weight curves of *PDE4A*^+/+^ and *PDE4A*^-/-^ mice fed HFD (n=9–10 mice per condition). (**E**) Body weight curves of *PDE4A^+/+^, PDE4A^-/-^*, *ob/ob* and *ob/ob*:*PDE4A^-/-^* mice fed ND during the first 10 weeks of age (n=7–10 mice per condition). **(F)** PDE4 activity in the cytosolic fraction of hypothalamus collected from *PDE4A^+/+^* and *PDE4A^-/-^* mice fed either ND or HFD (n=5–6 mice per group). All data in the figure are represented as mean ± SEM.

**Figure S4. HFD does not alter PDE4 activity in the amygdala, cortex, hippocampus or cerebellum.** (**A**) PDE4 activity in whole-hypothalamic lysates of amygdala collected from WT C57BL/6J mice fed either a normal diet (ND) or a high-fat diet (HFD) for 3 weeks (n=7–11 mice per condition). (**B**) PDE4 activity in the membrane and cytosolic fractions of amygdala collected from *PDE4A^+/+^* and *PDE4A^-/-^* mice fed either ND or HFD for 3 weeks (n=6 mice per condition). **(C–E)** PDE4 activity in whole-hypothalamic lysates collected from WT C57BL/6J mice fed either ND or HFD for 3 weeks. (**C**) cortical area (n=11 mice per group) (**D**) hippocampus (n=9–10 mice per group) and (**E**) cerebellum (n=9–11 mice per group). All data in the figure are represented as mean ± SEM.

**Figure S5. Genetic induced obesity, but not dietary induced obesity, alters total activity and anxiety levels of mice measured by the open field and elevated plus maze test.** (**A**) Open field test for *PDE4A^+/+^ and* *PDE4A^-/-^* mice fed either a normal diet (ND) or a high-fat diet (HFD) for 3 weeks; the *ob/ob* and *ob/ob*:PDE4A^-/-^ mice were fed ND (n=6–10 mice per condition; *P<0.05, ****P* < 0.001, *****P* < 0.0001, by one-way ANOVA). (**B**) Elevated plus maze test for PDE4A^+/+^and PDE4A^-/-^ mice fed either ND or HFD for 3 weeks; the *ob/ob* and *ob/ob*:*PDE4A^-/-^* mice were fed ND (n=3–12 mice per condition; *P<0.05, by one-way ANOVA). All data in the figure are represented as mean ± SEM.

**Figure S6. Free fatty acid receptors are regulated by either DIO or GIO.** Real-time PCR analysis of *FFAR3* (**A**) and *FFAR4* (**B**) in the hypothalamus of wild type (WT) C57BL/6J mice fed a normal diet (ND), WT C57BL/6J mice fed a high-fat diet (1 and 3 weeks), and *ob/ob* mice (n=4 mice per group, **P* < 0.05, by one-way ANOVA with Tukey’s multiple comparison test). (**C**) Membrane fractionation of mouse neuroblastoma cell line (N2a) treated with 500 mM of oleic acid at various time points. Phosphodiesterase 4A5 (Pde4a5) and β-arrestin2 (Arb2) levels normalized to total levels, respectively, were quantified by densitometry (Δ = fold changes). Two experiments with a representative blot are shown. All data in the figure are represented as mean ± SEM.
