# Supplementary material for: A high-fat diet promotes depression-like behavior in mice by suppressing hypothalamic PKA signaling
Source: Transl Psychiatry. 2019 May 10;9:141. doi: 10.1038/s41398-019-0470-1 (PMC6510753; doi:10.1038/s41398-019-0470-1)
Supplement: Supplementary file 2 — Supplementary Figure 1 [file 41398_2019_470_MOESM2_ESM.pptx]

## Slide 1
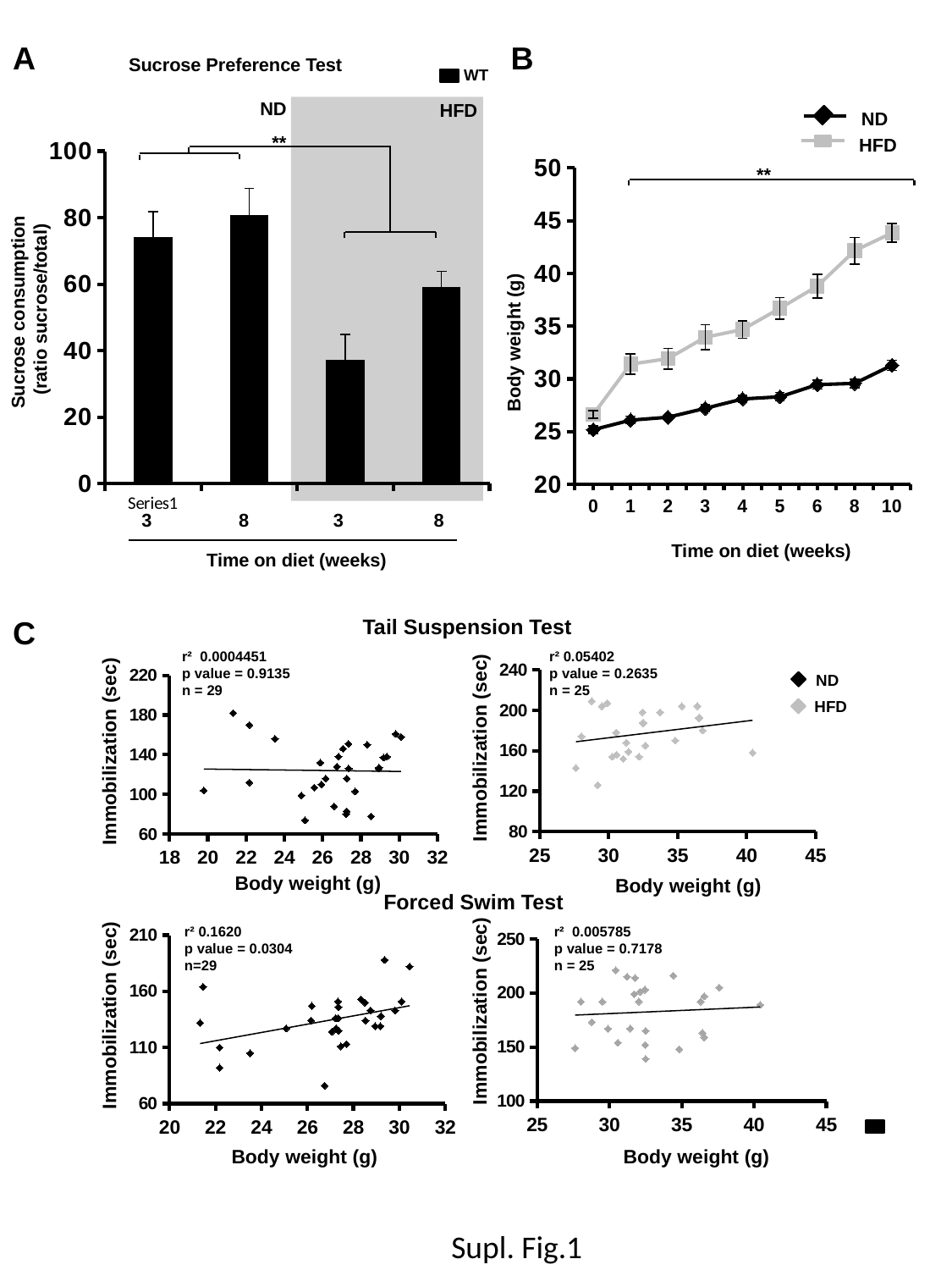

A
B
Sucrose Preference Test
WT
ND
HFD
ND
HFD
**
### Chart
| Category | |
|---|---|
| | 74.06432748538018 |
| | 80.8574187250657 |
| | 37.2863247863254 |
| | 59.07557720057753 |
### Chart
| Category | | |
|---|---|---|
| 0.0 | 25.183 | 26.633 |
| 1.0 | 26.08999999999999 | 31.39700000000001 |
| 2.0 | 26.362 | 31.91500000000001 |
| 3.0 | 27.20899999999999 | 33.94500000000001 |
| 4.0 | 28.099 | 34.68300000000001 |
| 5.0 | 28.30399999999999 | 36.692 |
| 6.0 | 29.456 | 38.792 |
| 8.0 | 29.572 | 42.14900000000001 |
| 10.0 | 31.29199999999999 | 43.83500000000001 |**
Sucrose consumption
(ratio sucrose/total)
Body weight (g)
8
3
3
8
Time on diet (weeks)
Time on diet (weeks)
C
Tail Suspension Test
r² 0.0004451
p value = 0.9135
n = 29
r² 0.05402
p value = 0.2635
n = 25
### Chart
| Category | |
|---|---|ND
HFD
### Chart
| Category | |
|---|---|Immobilization (sec)
Immobilization (sec)
Body weight (g)
Body weight (g)
Forced Swim Test
### Chart
| Category | |
|---|---|r² 0.005785
p value = 0.7178
n = 25
r² 0.1620
p value = 0.0304
n=29
### Chart
| Category | |
|---|---|Immobilization (sec)
Immobilization (sec)
Body weight (g)
Body weight (g)
Supl. Fig.1
