# Supplementary material for: A high-fat diet promotes depression-like behavior in mice by suppressing hypothalamic PKA signaling
Source: Transl Psychiatry. 2019 May 10;9:141. doi: 10.1038/s41398-019-0470-1 (PMC6510753; doi:10.1038/s41398-019-0470-1)
Supplement: Supplementary file 3 — Supplementary Figure 2 [file 41398_2019_470_MOESM3_ESM.pptx]

## Slide 1
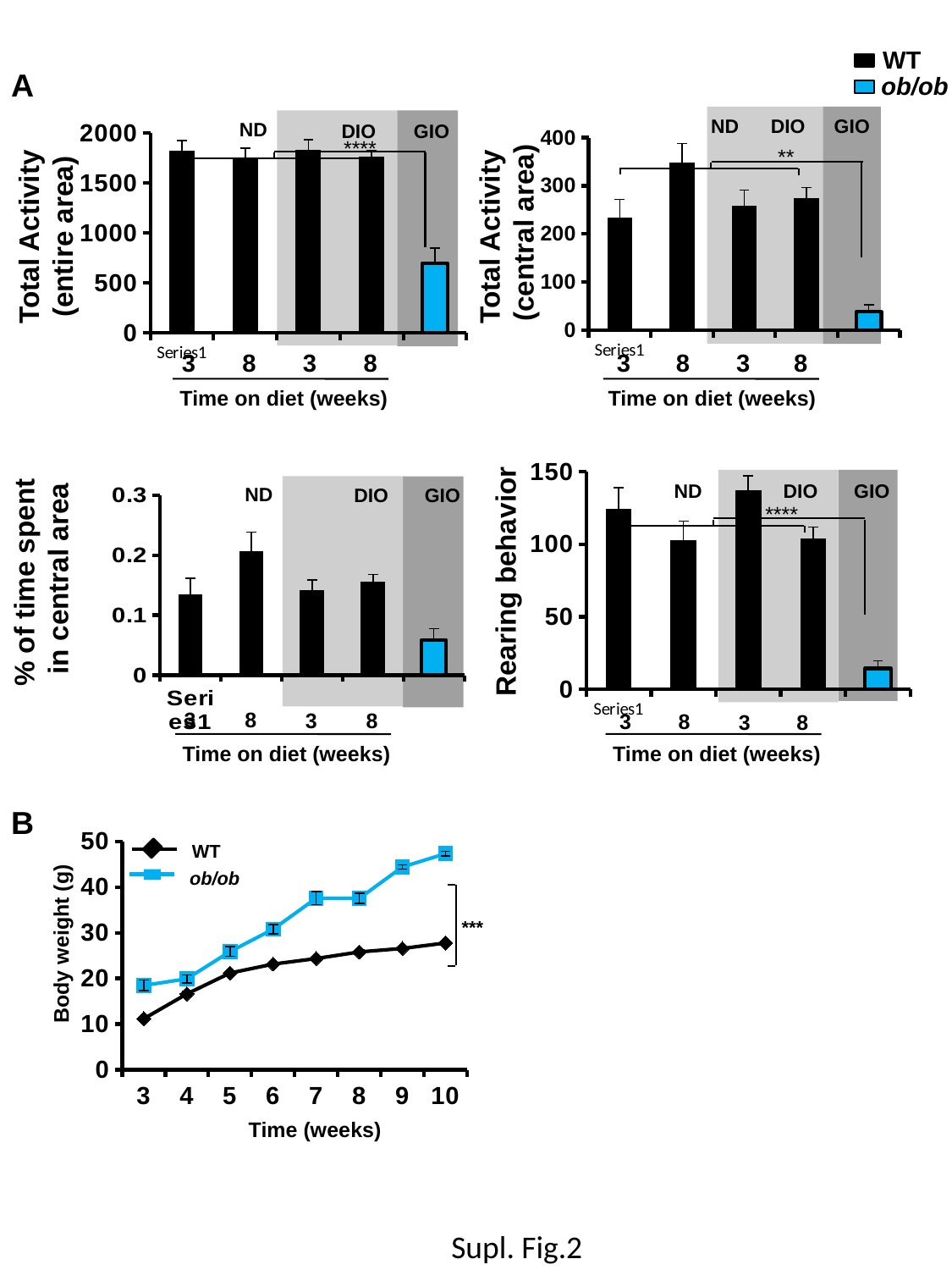

WT
A
ob/ob
ND
DIO
GIO
ND
DIO
GIO
### Chart
| Category | |
|---|---|
| | 1819.0 |
| | 1750.7 |
| | 1833.4 |
| | 1764.5 |
| | 694.5714285714286 |
### Chart
| Category | |
|---|---|
| | 234.5 |
| | 348.8 |
| | 257.8999999999999 |
| | 273.8 |
| | 38.42857142857143 |****
**
Total Activity
(entire area)
Total Activity
 (central area)
3
8
3
8
3
8
3
8
Time on diet (weeks)
Time on diet (weeks)
### Chart
| Category | |
|---|---|
| | 124.2 |
| | 103.0 |
| | 137.4 |
| | 103.8 |
| | 14.42857142857143 |
ND
DIO
GIO
### Chart
| Category | |
|---|---|
| | 0.135079639 |
| | 0.206965385718105 |
| | 0.141484574 |
| | 0.155266667976576 |
| | 0.0585757648902152 |ND
DIO
GIO
****
% of time spent
in central area
Rearing behavior
8
3
3
8
8
3
3
8
Time on diet (weeks)
Time on diet (weeks)
B
### Chart
| Category | ob/ob | wt |
|---|---|---|
| 3.0 | 18.45 | 11.17666666666667 |
| 4.0 | 19.9 | 16.5953333333333 |
| 5.0 | 25.86666666666666 | 21.15533333333329 |
| 6.0 | 30.76666666666667 | 23.13066666666667 |
| 7.0 | 37.55 | 24.346 |
| 8.0 | 37.51666666666659 | 25.78266666666666 |
| 9.0 | 44.38333333333333 | 26.5513333333333 |
| 10.0 | 47.28333333333334 | 27.75099999999999 |WT
ob/ob
***
Body weight (g)
Time (weeks)
Supl. Fig.2
