# Supplementary material for: A high-fat diet promotes depression-like behavior in mice by suppressing hypothalamic PKA signaling
Source: Transl Psychiatry. 2019 May 10;9:141. doi: 10.1038/s41398-019-0470-1 (PMC6510753; doi:10.1038/s41398-019-0470-1)
Supplement: Supplementary file 4 — Supplementary Figure 3 [file 41398_2019_470_MOESM4_ESM.pptx]

## Slide 1
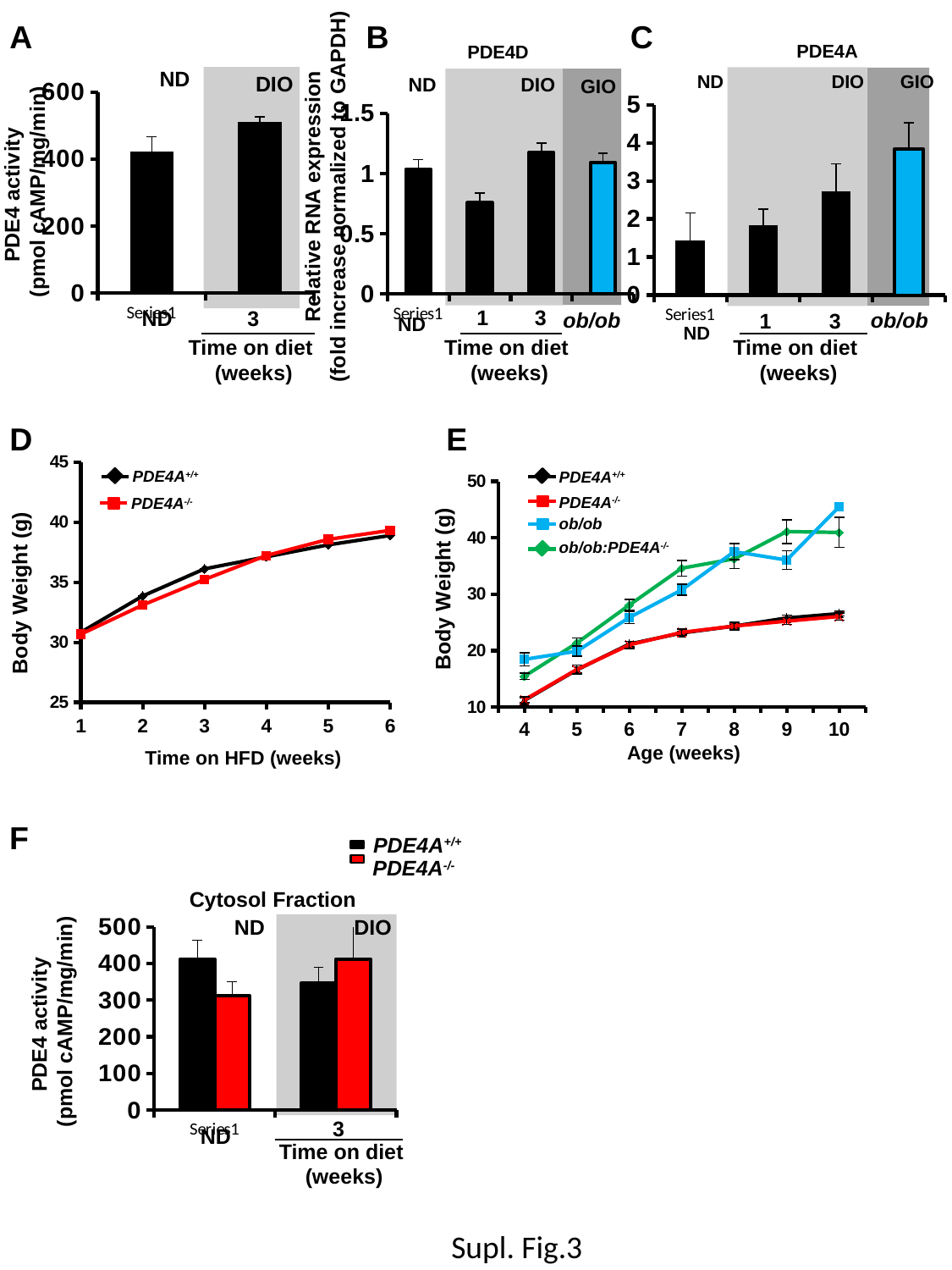

A
B
C
PDE4A
PDE4D
ND
ND
DIO
GIO
DIO
ND
DIO
GIO
### Chart
| Category | |
|---|---|
| | 422.7508527008804 |
| | 510.9815182041716 |
### Chart
| Category | |
|---|---|
| | 1.427605139384069 |
| | 1.832941085103318 |
| | 2.717670216257446 |
| | 3.839020695046277 |
### Chart
| Category | |
|---|---|
| | 1.034666239031698 |
| | 0.756965747823279 |
| | 1.173817495701408 |
| | 1.090086784268595 |PDE4 activity
(pmol cAMP/mg/min)
Relative RNA expression
(fold increase normalized to GAPDH)
1
3
3
ND
ob/ob
ob/ob
3
1
ND
ND
Time on diet
(weeks)
Time on diet
(weeks)
Time on diet
(weeks)
D
E
### Chart
| Category | pde4a+/+ | pde4a-/- |
|---|---|---|PDE4A+/+
PDE4A+/+
PDE4A-/-
ob/ob
ob/ob:PDE4A-/-
### Chart
| Category | pde4a-/- ob/ob | ob/ob | PDE4A +/+ | PDE4A-/- |
|---|---|---|---|---|
| 4.0 | 15.45192307692307 | 18.45 | 11.17666666666667 | 11.26692307692307 |
| 5.0 | 21.3414814814815 | 19.9 | 16.5953333333333 | 16.65384615384616 |
| 6.0 | 28.08607142857143 | 25.86666666666666 | 21.15533333333329 | 21.02307692307692 |
| 7.0 | 34.60703703703702 | 30.76666666666667 | 23.13066666666667 | 23.2276923076923 |
| 8.0 | 36.26263157894737 | 37.55 | 24.346 | 24.35153846153847 |
| 9.0 | 41.1036842105263 | 36.05 | 25.78266666666666 | 25.21769230769231 |
| 10.0 | 40.94538461538457 | 45.5 | 26.5513333333333 | 26.04692307692308 |
PDE4A-/-
Body Weight (g)
Body Weight (g)
Age (weeks)
Time on HFD (weeks)
F
PDE4A+/+
PDE4A-/-
Cytosol Fraction
### Chart
| Category | | |
|---|---|---|
| | 411.9026026306742 | 312.0185088020839 |
| | 346.1756617394293 | 411.922278410016 |ND
DIO
PDE4 activity
(pmol cAMP/mg/min)
3
ND
Time on diet
(weeks)
Supl. Fig.3
