# Supplementary material for: A high-fat diet promotes depression-like behavior in mice by suppressing hypothalamic PKA signaling
Source: Transl Psychiatry. 2019 May 10;9:141. doi: 10.1038/s41398-019-0470-1 (PMC6510753; doi:10.1038/s41398-019-0470-1)
Supplement: Supplementary file 5 — Supplementary Figure 4 [file 41398_2019_470_MOESM5_ESM.pptx]

## Slide 1
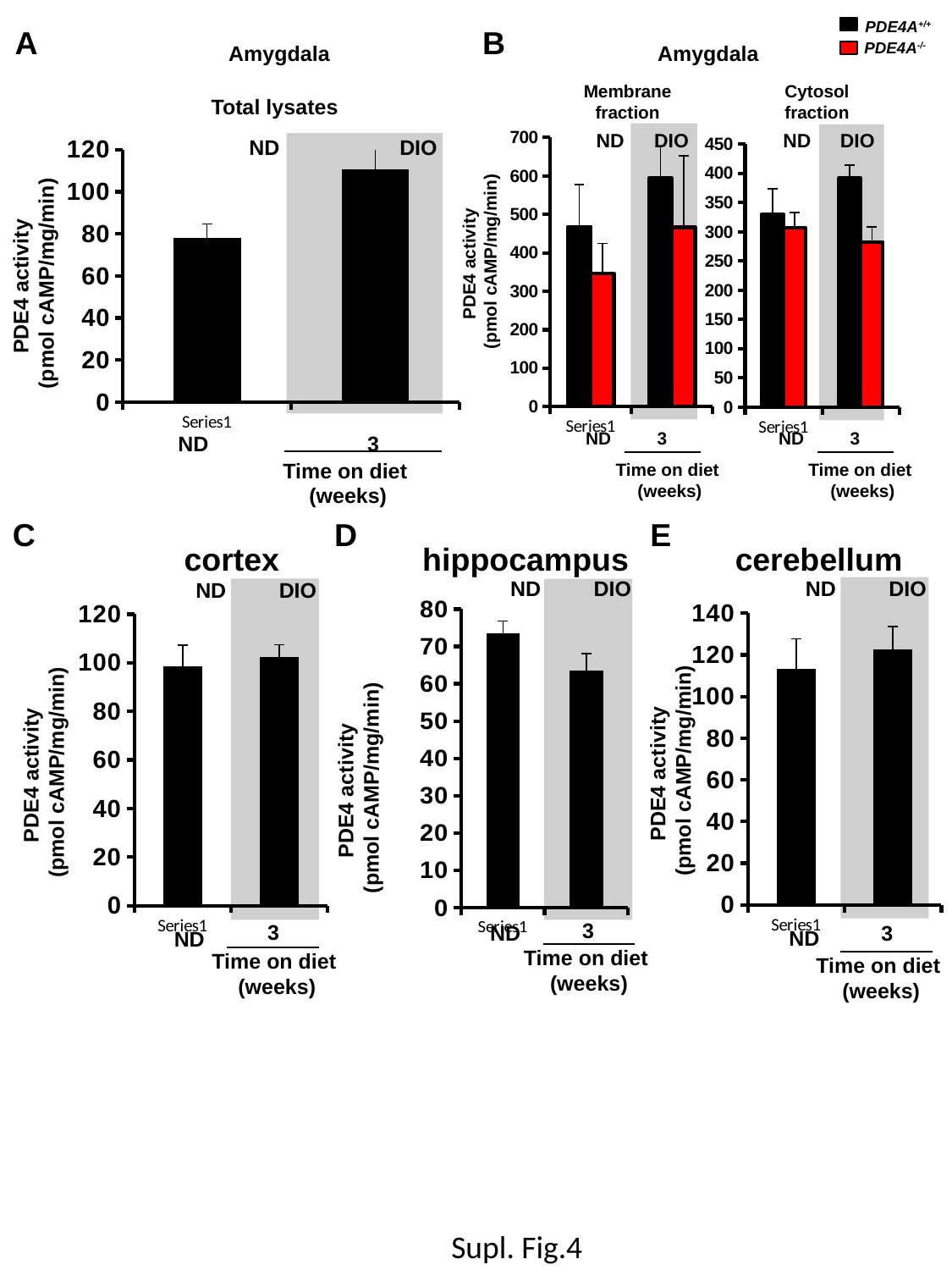

PDE4A+/+
A
B
PDE4A-/-
Amygdala
Amygdala
Membrane
fraction
Cytosol
fraction
Total lysates
### Chart
| Category | | |
|---|---|---|
| | 468.3049999549312 | 346.6558801525134 |
| | 593.8261102748304 | 466.5503510875146 |ND
DIO
ND
DIO
ND
DIO
### Chart
| Category | | |
|---|---|---|
| | 329.742835254379 | 306.6802764912769 |
| | 391.8993975693841 | 282.8161454887346 |
### Chart
| Category | |
|---|---|
| | 77.8974272952175 |
| | 110.572704125963 |
PDE4 activity
 (pmol cAMP/mg/min)
PDE4 activity
(pmol cAMP/mg/min)
3
3
ND
ND
ND
3
Time on diet
(weeks)
Time on diet
(weeks)
Time on diet
(weeks)
C
D
E
cortex
hippocampus
cerebellum
ND
DIO
ND
DIO
ND
DIO
### Chart
| Category | |
|---|---|
| | 73.49007453799747 |
| | 63.623529371622 |
### Chart
| Category | |
|---|---|
| | 113.4484399796192 |
| | 122.7944081324575 |
### Chart
| Category | |
|---|---|
| | 98.59703334641851 |
| | 102.2954080757242 |PDE4 activity
(pmol cAMP/mg/min)
PDE4 activity
(pmol cAMP/mg/min)
PDE4 activity
(pmol cAMP/mg/min)
3
3
ND
3
ND
ND
Time on diet
(weeks)
Time on diet
(weeks)
Time on diet
(weeks)
Supl. Fig.4
