# Supplementary material for: A high-fat diet promotes depression-like behavior in mice by suppressing hypothalamic PKA signaling
Source: Transl Psychiatry. 2019 May 10;9:141. doi: 10.1038/s41398-019-0470-1 (PMC6510753; doi:10.1038/s41398-019-0470-1)
Supplement: Supplementary file 6 — Supplementary Figure 5 [file 41398_2019_470_MOESM6_ESM.pptx]

## Slide 1
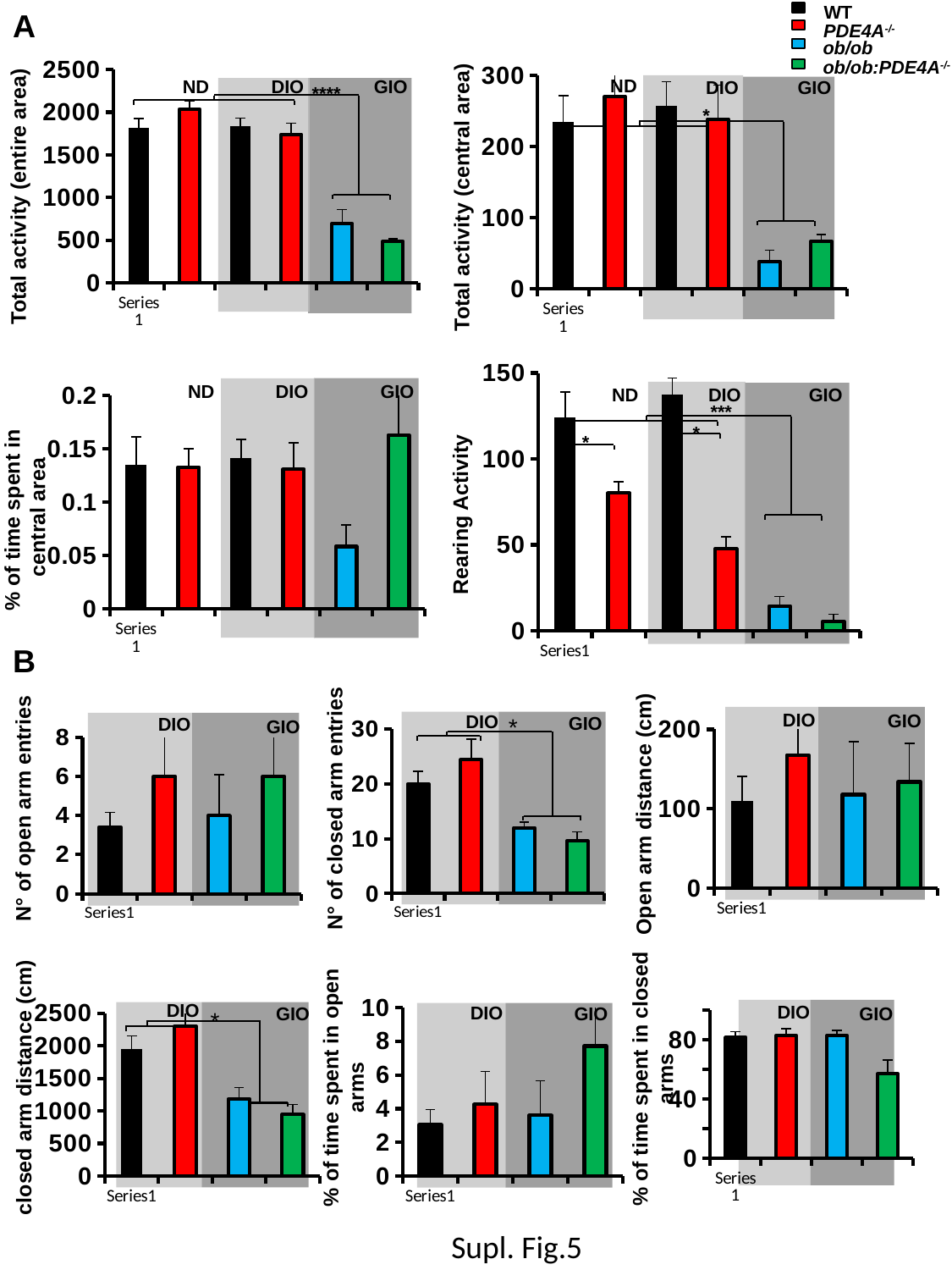

A
WT
PDE4A-/-
ob/ob
ob/ob:PDE4A-/-
### Chart
| Category | |
|---|---|
| | 1819.0 |
| | 2035.571428571428 |
| | 1833.4 |
| | 1739.166666666667 |
| | 694.5714285714286 |
| | 487.2222222222222 |
### Chart
| Category | |
|---|---|
| | 234.5 |
| | 270.888888888889 |
| | 257.8999999999999 |
| | 238.875 |
| | 38.42857142857143 |
| | 67.11111111111111 |ND
ND
DIO
GIO
DIO
GIO
****
*
Total activity (entire area)
Total activity (central area)
### Chart
| Category | |
|---|---|
| | 124.2 |
| | 80.11111111111111 |
| | 137.4 |
| | 47.625 |
| | 14.42857142857143 |
| | 5.444444444444445 |ND
DIO
GIO
### Chart
| Category | |
|---|---|
| | 0.135079639 |
| | 0.132998938888889 |
| | 0.141484574 |
| | 0.13101268125 |
| | 0.0585757648902152 |
| | 0.163005403545412 |ND
DIO
GIO
***
*
*
% of time spent in
 central area
Rearing Activity
B
### Chart
| Category | |
|---|---|
| | 3.4 |
| | 6.0 |
| | 4.0 |
| | 6.0 |DIO
GIO
DIO
GIO
*
DIO
GIO
### Chart
| Category | |
|---|---|
| | 110.0 |
| | 167.6 |
| | 118.0 |
| | 133.9166666666666 |
### Chart
| Category | |
|---|---|
| | 20.0 |
| | 24.4 |
| | 12.0 |
| | 9.583333333333336 |N° of open arm entries
N° of closed arm entries
Open arm distance (cm)
### Chart
| Category | |
|---|---|
| | 3.043333333333334 |
| | 4.256666666666666 |
| | 3.605555555555556 |
| | 7.722222222222221 |DIO
DIO
DIO
### Chart
| Category | |
|---|---|
| | 1957.4 |
| | 2303.0 |
| | 1184.0 |
| | 944.75 |GIO
GIO
GIO
*
### Chart
| Category | |
|---|---|
| | 81.72800000000001 |
| | 82.96860000000002 |
| | 82.88888888888879 |
| | 56.9625 |% of time spent in closed arms
% of time spent in open arms
closed arm distance (cm)
Supl. Fig.5
