# Supplementary material for: A high-fat diet promotes depression-like behavior in mice by suppressing hypothalamic PKA signaling
Source: Transl Psychiatry. 2019 May 10;9:141. doi: 10.1038/s41398-019-0470-1 (PMC6510753; doi:10.1038/s41398-019-0470-1)
Supplement: Supplementary file 7 — Supplementary Figure 6 [file 41398_2019_470_MOESM7_ESM.pptx]

## Slide 1
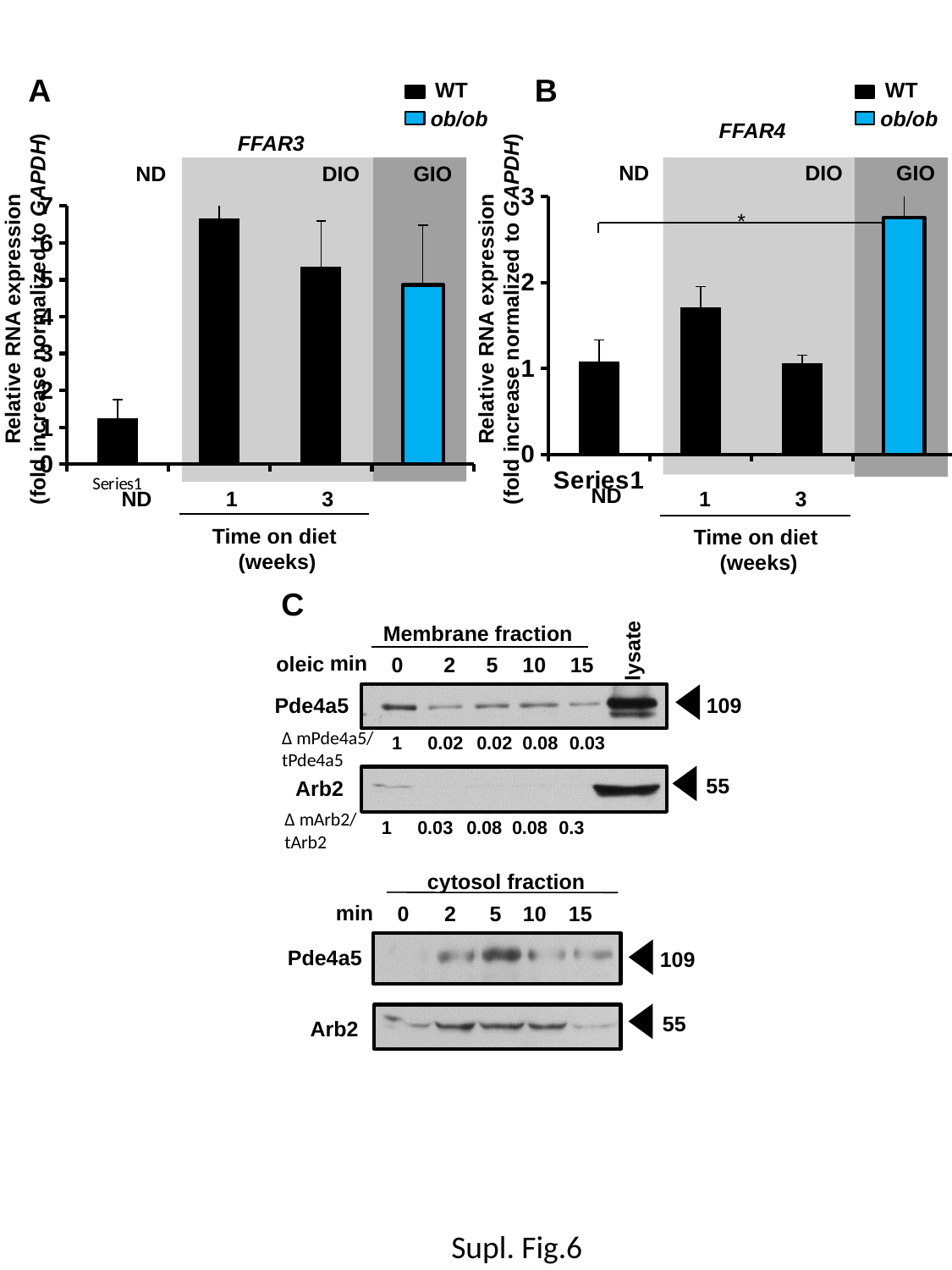

A
B
WT
ob/ob
WT
ob/ob
FFAR4
FFAR3
ND
DIO
GIO
ND
DIO
GIO
### Chart
| Category | |
|---|---|
| | 1.08207403933238 |
| | 1.707344314595113 |
| | 1.055497093092778 |
| | 2.757536083983571 |
### Chart
| Category | |
|---|---|
| | 1.245202932 |
| | 6.659677899 |
| | 5.358382460999996 |
| | 4.861259528999996 |*
Relative RNA expression
(fold increase normalized to GAPDH)
Relative RNA expression
(fold increase normalized to GAPDH)
ND
ND
1
3
1
3
Time on diet
(weeks)
Time on diet
(weeks)
C
Membrane fraction
lysate
min
oleic
0
2
5
10
15
Pde4a5
109
Δ mPde4a5/
tPde4a5
1
0.02
0.02
0.08
0.03
55
Arb2
Δ mArb2/
tArb2
1
0.03
0.08
0.08
0.3
cytosol fraction
min
0
2
5
10
15
Pde4a5
109
55
Arb2
Supl. Fig.6
